# The Ability of Near-Infrared Autofluorescence to Protect Parathyroid Gland Function During Thyroid Surgery: A Meta-Analysis

**DOI:** 10.3389/fendo.2021.714691

**Published:** 2021-10-25

**Authors:** Bin Wang, Chun-Rong Zhu, Hong Liu, Xin-Min Yao, Jian Wu

**Affiliations:** ^1^ Department of Thyroid and Breast Surgery, Chengdu Third People’s Hospital, Chengdu, China; ^2^ College of Preclinical Medicine, North Sichuan Medical College, Nanchong, China

**Keywords:** near-infrared, autofluorescence, parathyroid gland function, thyroid surgery, meta-analysis

## Abstract

**Objective:**

We conducted this meta-analysis to assess the ability of near-infrared autofluorescence to protect parathyroid gland function during thyroid surgery.

**Method:**

A systematic literature search was conducted using PubMed, Embase, and the Cochrane Library electronic databases for studies published up to February 2021. The reference lists of the retrieved articles were also reviewed. Two authors independently assessed methodological quality and extracted the data. A random-effects model was used to calculate the overall pooled variable and the weighted mean deviation. Publication bias in these studies was evaluated using the Egger’s and Begg’s tests.

**Result:**

Seven studies involving 1,480 patients were included in the analysis. Compared with patients in the naked eye group, the pooled relative risk of inadvertent parathyroid gland resection and parathyroid gland autotransplantation for the patients in the near-infrared autofluorescence group was 0.48 (95% CI, 0.26–0.9, p = 0.023) and 0.39 (95% CI, 0.09–1.68, p = 0.208), respectively. The pooled relative risk of hypocalcemia at 1 day postoperatively and at 6 months postoperatively for the patients in the near-infrared autofluorescence group was 0.49 (95% CI, 0.34–0.71, p < 0.001) and 0.34 (95% CI, 0.06–2.03, p = 0.238) compared with patients in the naked eye group.

**Conclusion:**

Near-infrared autofluorescence is significantly associated with a reduced risk of inadvertent parathyroid gland resection and hypocalcemia at 1 day postoperatively.

## Introduction

Thyroidectomy with or without lymph node dissection is the primary therapeutic method for thyroid neoplasms (1, 2). Hypoparathyroidism is a frequent complication after thyroid surgery, occurring in 33.7%–49% of patients and remains permanent in 6%–19.4% of patients (3–5). Postoperative hypoparathyroidism is the result of parathyroid gland (PG) damage, devascularization, and inadvertent resection. This complication can lead to poor experience, prolonged hospitalization, and increased costs (6, 7). Permanent hypoparathyroidism requires long-time substitutive treatment, impairs immune function, and thus lowers quality of life (8–10).

Although some strategies have been proposed to prevent postoperative hypoparathyroidism, such as meticulous capsule dissection, preservation of the inferior thyroid vein, and classification of PG for functional protection, PG identification is crucial during thyroid surgery (11–14). Several tracers were reported to assist in identifying the PG, but they have some disadvantages, such as lack of direct evidence and limitation of instrument and/or invasiveness, and surgeon-dependent identification is still pivotal after using them (11, 15, 16).

In recent years, near-infrared autofluorescence (NIRAF) has been introduced to identify PG during surgery (17–19). Several studies investigated the ability of NIRAF to protect PG function and confirmed that it helped to reduce the number of inadvertently resected PGs and the incidence of postoperative transient hypocalcemia (14, 20). However, DiMarco et al. (21) reported that the method did not significantly reduce PG in postoperative specimens, and Papavramidis et al. (22) suggested that the method did not reduce the incidence of postoperative hypoparathyroidism and hypocalcemia. Due to the disagreement over the protective ability, we conducted a meta-analysis to assess the ability of NIRAF to protect PG function.

## Methods

### Literature Search

The initial search criteria were defined in terms of populations, interventions, comparators, outcomes, and study designs (PICOS) as follows: P, patients who underwent thyroid surgery; I, near-infrared autofluorescence of parathyroid gland; C, white light or naked eyes; O, the number of identified parathyroid, the incidence of inadvertently resected parathyroid gland, the incidence of parathyroid gland autotransplantation, and the incidence of postoperative hypoparathyroidism or hypocalcemia after total thyroidectomy; and S, no restrictions. Because only few studies were obtained in preliminary research, we made the research strategy according to the interventions.

Two investigators independently conducted a search using PubMed, Embase, and the Cochrane Library electronic databases for studies published up to February 28, 2021. The search algorithm was [(near-infrared) AND (parathyroid)] for PubMed. The following search terms were used in all fields as a search strategy for Embase: (1) parathyroid glands, parathyroid gland, parathyroid, parathyroids; (2) (spectroscopy, near-infrared), near-infrared spectroscopy, near infrared spectroscopy; near infrared, near-infrared. For Cochrane Library electronic databases, the search strategy was the following terms by searching Medical Subject Headings and free word in all field: (1) parathyroid glands, parathyroid gland, parathyroid, parathyroids; (2) (spectroscopy, near-infrared), near infrared spectroscopy, near infrared spectroscopies, near infra-red spectroscopy, near infra-red spectroscopies, near infrared, near infra-red. No restrictions were imposed. In addition, we reviewed the reference lists of the retrieved papers and recent reviews.

### Study Selection

The first screening of each paper was performed based on the title and abstract, and the full text was then reviewed. Studies were considered eligible if they met all the following criteria (1): the exposure of interest included NIRAF (2); the outcome of interest was the number of identified PG, the incidence of inadvertently resected PG or PG autotransplantation, and/or the incidence of hypoparathyroidism and/or hypocalcemia; and (3) relative risk (RR) and the corresponding 95% confidence interval (CI) (or data to calculate these values) were reported. Studies were excluded based on the following criteria: (1) conference abstract, review, case report, commentary, discussion, and letter; (2) those in which the fluorescence originated from tracer or if angiography was used; (3) those in which the trial was not conducted in humans; (4) those which were published in non-English languages; and (5) those from which data could not be collected adequately.

### Data Extraction and Quality Assessment

Data were extracted by two reviewers (BW and C-RZ) using a predefined data extraction form. Data were collected as follows: first author, publication date, type of study, country of origin, study sites and institutes, measurement instrument and method, research period, sample size, disease (the reason for surgery) and surgical method, the number of cases and controls, the number and/or the mean number and the standard deviation (SD) of identified PG, incidence of inadvertently resected PG and/or PG autotransplantation, evaluation indexes of hypoparathyroidism and hypocalcemia, and the incidence of hypoparathyroidism or hypocalcemia after surgery. The quality of randomized controlled trials was assessed using the critical appraisal skills program (CASP) checklist (23). The quality of cohort studies and case–control studies was assessed using the Newcastle–Ottawa Scale (NOS), and studies with an NOS score >5 were considered high-quality studies (24). The quality of non-randomized studies was assessed using the methodological index for non-randomized studies (MINORS) (25). BW and C-RZ independently conducted the study selection, data extraction, and quality assessment. All disagreements in these processes were discussed and resolved by consensus.

### Statistical Analysis

Stata version 14.0 (Stata Corp LP, College Station, TX, USA) was used to calculate the pooled relative risk (RR), pooled odds ratio (OR), and weighted mean deviation (WMD) with a random-effects model (DerSimonian–Laird). Heterogeneity was quantified using the I^2^ test. p< 0.1 and I^2^ > 50% for heterogeneity were considered significant differences. Potential publication bias was assessed using the Begg rank correlation test (26) and the Egger linear regression test (27). p < 0.05 was considered statistically significant in all tests.

## Results

### Literature Search

**Figure 1:**
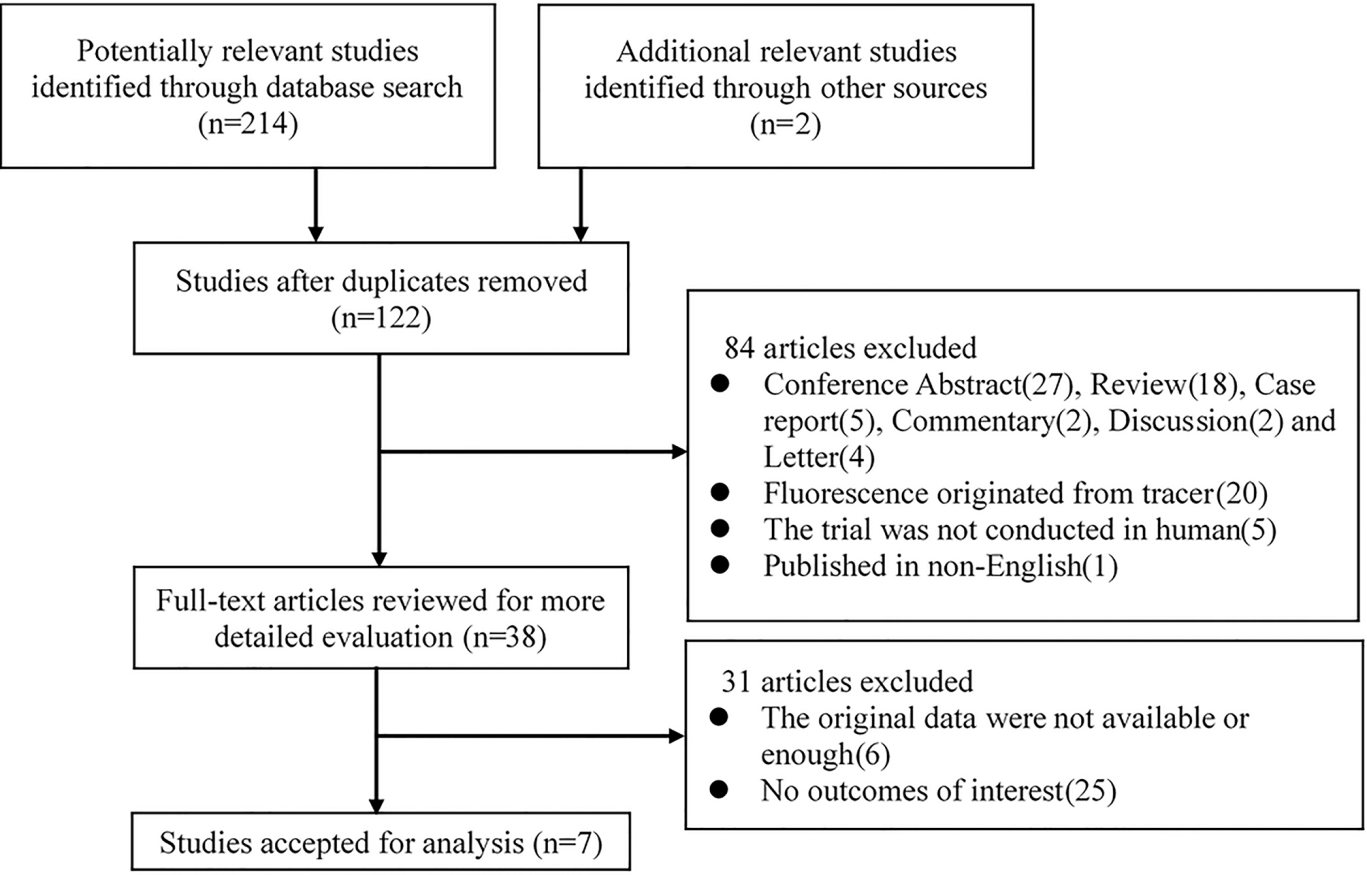
Flow chart of study selection.

The study selection process is illustrated in **Figure 1**. A total of 214 potentially relevant records were identified by searching the abovementioned databases, and two other records were added by reviewing the reference lists of the retrieved papers. One hundred twenty-two records were retained after duplicates were removed. After screening the titles and abstracts, 84 studies were excluded according to the exclusion criteria. The remaining 38 studies were assessed *via* full-text screening, and 31 studies were further excluded. Finally, seven independent studies were included in the meta-analysis (14, 20–22, 28–30).

### Study Characteristics

**Table 1** shows the basic information of the seven eligible studies (14, 20–22, 28–30). All of the studies were published in recent 4 years and were prospective studies. Of the seven studies, three were randomized control trials, one was a prospective cohort study, one was a prospective matched case–control study, one was a historical controlled study, and one was a before–after study in the same patient. Four, one, and two studies were conducted in Europe, the United States, and Argentina, respectively. One study that used both NIRAF and angiography and consisted of thyroid disease and primary hyperparathyroidism was also included because of the detailed information of the surgical process and the outcome of interest, which was the number of identified PGs (28). According to these studies, two types of instruments based on the NIRAF were used to identify PGs, and a total of 1,480 patients were included in the analysis. The outcome of interest varied across studies, including the number of identified PGs, the incidence of inadvertently resected PG and/or PG autotransplantation, and the incidence of hypoparathyroidism or hypocalcemia. According to the CASP lists, NOS, and MINORs, all of the included studies demonstrated relatively high quality.

**Table 1:** The characteristics of included studies.

Study ID	First author	Publication date	Type of study	Publication quality (method: score/total points)	Country/region	Institute	Instrument	Measurement method	Research period	Patients (NIRAF/control)	Disease	Surgical method	Golden standard	Follow-up time	Outcome of interest
**1**	Falco	2017	Before–after	Minors: 18/24	Argentina	University of Buenos Aires	Fluobeam (Fluoptics, Grenoble, France)	20 cm	2015.10–2016.2	74/74	T, pHPT	Tx, pPTx	Experience, pathology reports	1d	IPG
**2**	Benmiloud	2018	Historical controlled study	NOS: 9/9	France	Hôpital Européen Marseille	Fluobeam (Fluoptics, Grenoble, France)	15–20 cm	2015.1–2016.9	93/153	T	TT	Experience	6m	IPG, IRPG, PGA, hypocalcemia (<8.0 mg/dL)
**3**	DiMarco	2019	PC	NOS: 7/9	UK	Hammersmith Hospital, Imperial College	Fluobeam (Fluoptics, Grenoble, France)	20 cm	2016.1–2017.10	106/163	T	Tx	Experience, Pathology reports	6m	IRPG, hypocalcemia (<2 mmol/l)
**4**	Dip	2019	RCT	CASP RCT Checklist	Argentina	Instituto Argentino de Diagnosticoy Tratamiento	Fluobeam (Fluoptics, Grenoble, France)	20cm	2017.1–2017.8	85/85	T	TTx	Experience, Pathology reports	1d	IPG, hypocalcemia (<8.0 mg/dl)
**5**	Benmiloud	2020	RCT	CASP RCT Checklist	France	Hôpital Européen Marseille, Groupement Hospitalier Universitaire Pitié Salpêtrière, Hôpital Saint-Joseph	Fluobeam (Fluoptics, Grenoble, France)	20 cm	2016.9–2018.10	121/120	T	TTx	Experience, Pathology reports	6m	IPG, IRPG, PGA, hypocalcemia (<8.0 mg/dl)
**6**	Kim	2020	Prospective matched case–control	NOS: 8/9	American	Cleveland Clinic, Cleveland, Ohio	Fluobeam (Fluoptics, Grenoble, France)	20 cm	2012.12–2019.10	100/200	T	TTx	Experience, pathology reports	6m	IPG, IRPG, PGA, hypocalcemia (<8.0 mg/dl)
**7**	Papavramidis	2021	RCT	CASP RCT Checklist	Greece	AHEPA University Hospital; Interbalkan Medical Center	Fluobeam LX (Fluoptics, Grenoble, France)	20 cm	2019.12–2020.3	90/90	T	TTx	Experience, pathology reports	1d	IRPG, hypocalcemia (<8.0 mg/dl)

Before–after, before–after study in the same patient; PC, prospective cohort study; RCT, randomized controlled trial; MINORS, methodological index for non-randomized studies; NOS, Newcastle–Ottawa Scale; CASP, critical appraisal skills program; T thyroid disease, pHPT primary hyperparathyroidism; Tx thyroidectomy (hemi- or total); TTx, total thyroidectomy; PTx, parathyroidectomy; IPG, identified parathyroid gland; IRPG, inadvertently resected parathyroid gland; PGA, parathyroid gland autotransplantation.

### The Ability of NIRAF in Protecting PG

**Figure 2:**
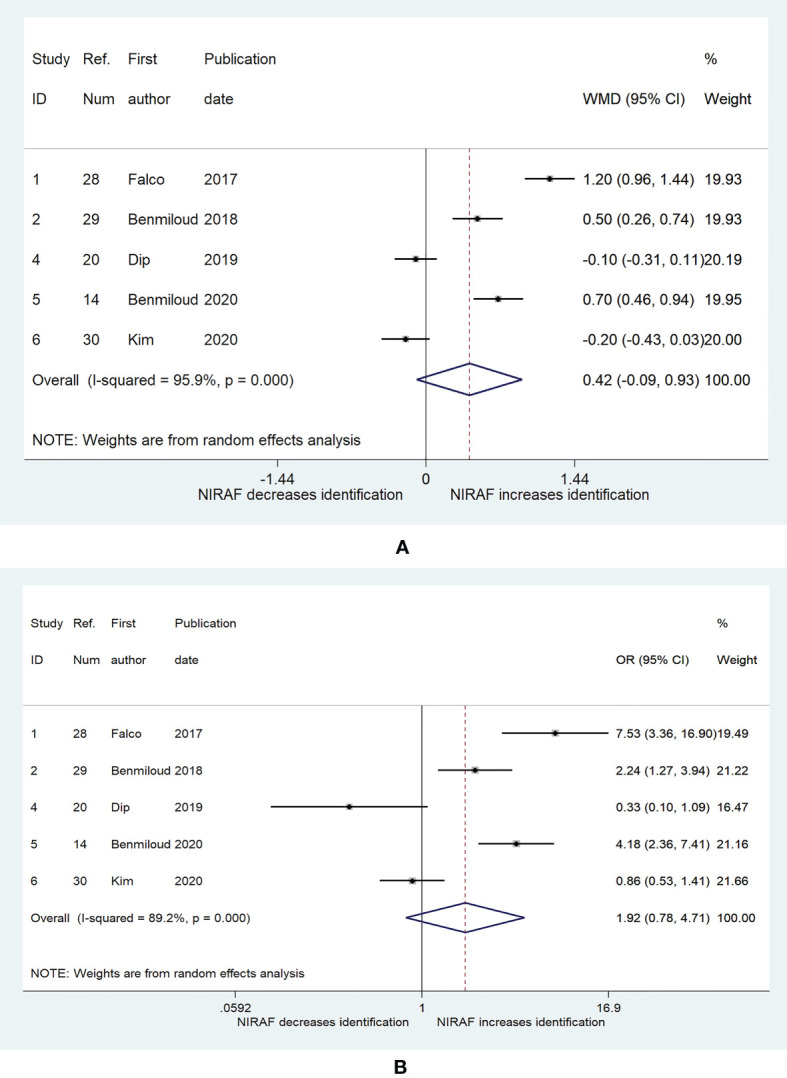
The relationship between near-infrared autofluorescence and the number of identified PG. **(A)** the weighted mean deviation of the number of identified parathyroid between near-infrared autofluorescence group and naked eye group. **(B)** the odds ratio (patients with three or more identified parathyroid glands vs. patients with two or less identified parathyroid glands) between near-infrared autofluorescence group and naked eye group. The shaded areas represent the weight of the relevant study in pooling the results; the error bars represent the 95% confidence interval.

Five studies explored the relationship between NIRAF and the number of identified PGs (14, 20, 28–30). The WMD was 0.42 (95% CI, −0.09–0.93, p = 0.107, **Figure 2A**), while the heterogeneity was significant (I^2^ = 95.9%, p < 0.001, **Figure 2A**). The publication bias, as measured by Begg’s and Egger’s tests, did not appear to be significant (p = 0.221, p = 0.205). We classified the participants according to whether the number of identified PGs was more than 2. The pooled OR was 1.92 (95% CI, 0.78–4.71, p = 0.153, **Figure 2B**), and the heterogeneity was still significant (I^2^ = 89.2%, p < 0.001, **Figure 2B**). The publication bias was not significant (Begg, p = 0.462; Egger, p = 0.988).

**Figure 3:**
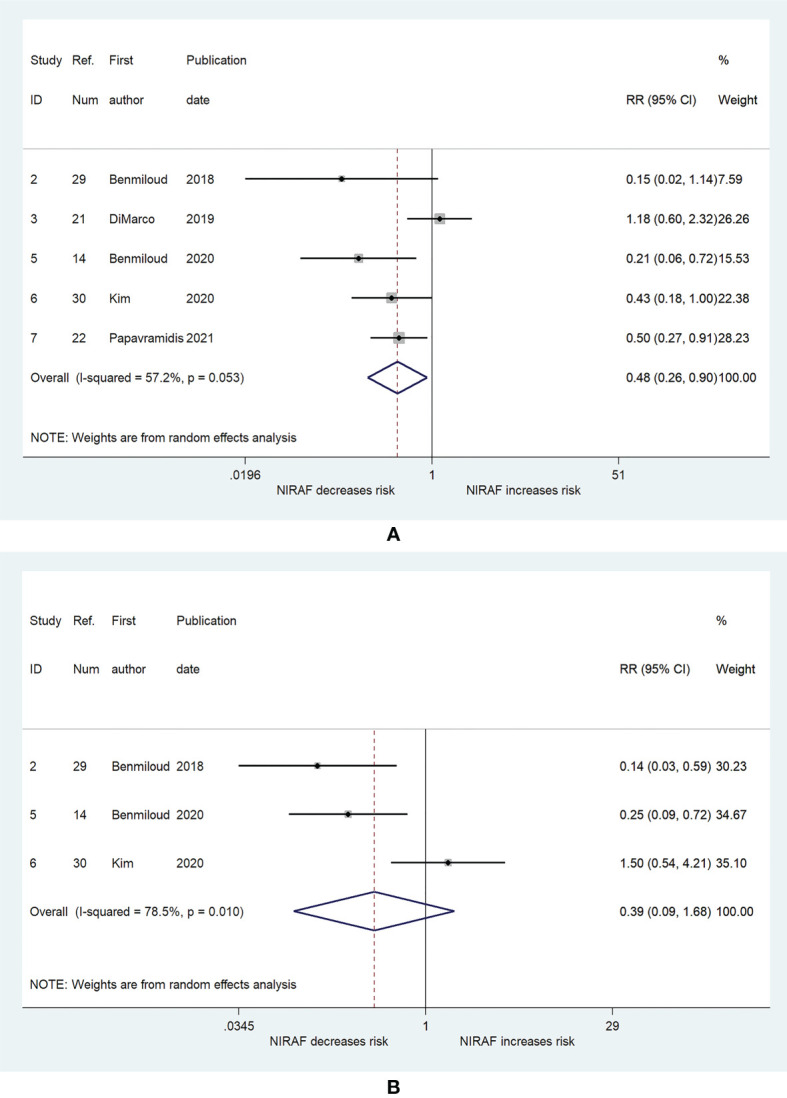
The relative risk for inadvertent parathyroid gland resection and autotransplantation. **(A)** Inadvertent parathyroid gland resection. **(B)** parathyroid gland autotransplantation. The shaded areas represent the weight of the relevant study in pooling the results; the error bars represent the 95% confidence interval.

**Figure 3A** shows the results of the pooled RR for the risk of inadvertently resected PG. Five studies (14, 21, 22, 29, 30) were included in the analysis. The RRs for the relationship between NIRAF and inadvertently resected PG varied from 0.15 to 1.18 across the studies, while the pooled RR was 0.48 (95% CI, 0.26–0.9, p = 0.023). The heterogeneity was moderate ((I^2^ = 57.2%, p = 0.053), and the publication bias was not significant (Begg, p = 0.221; Egger, p = 0.219). **Figure 3B** presents the pooled RR related to NIRAF and PG autotransplantation. NIRAF was not associated with the incidence of PG autotransplantation (RR = 0.39; 95% CI, 0.09–1.68, p = 0.208; Begg, p = 0.296; Egger, p = 0.521).

**Figure 4:**
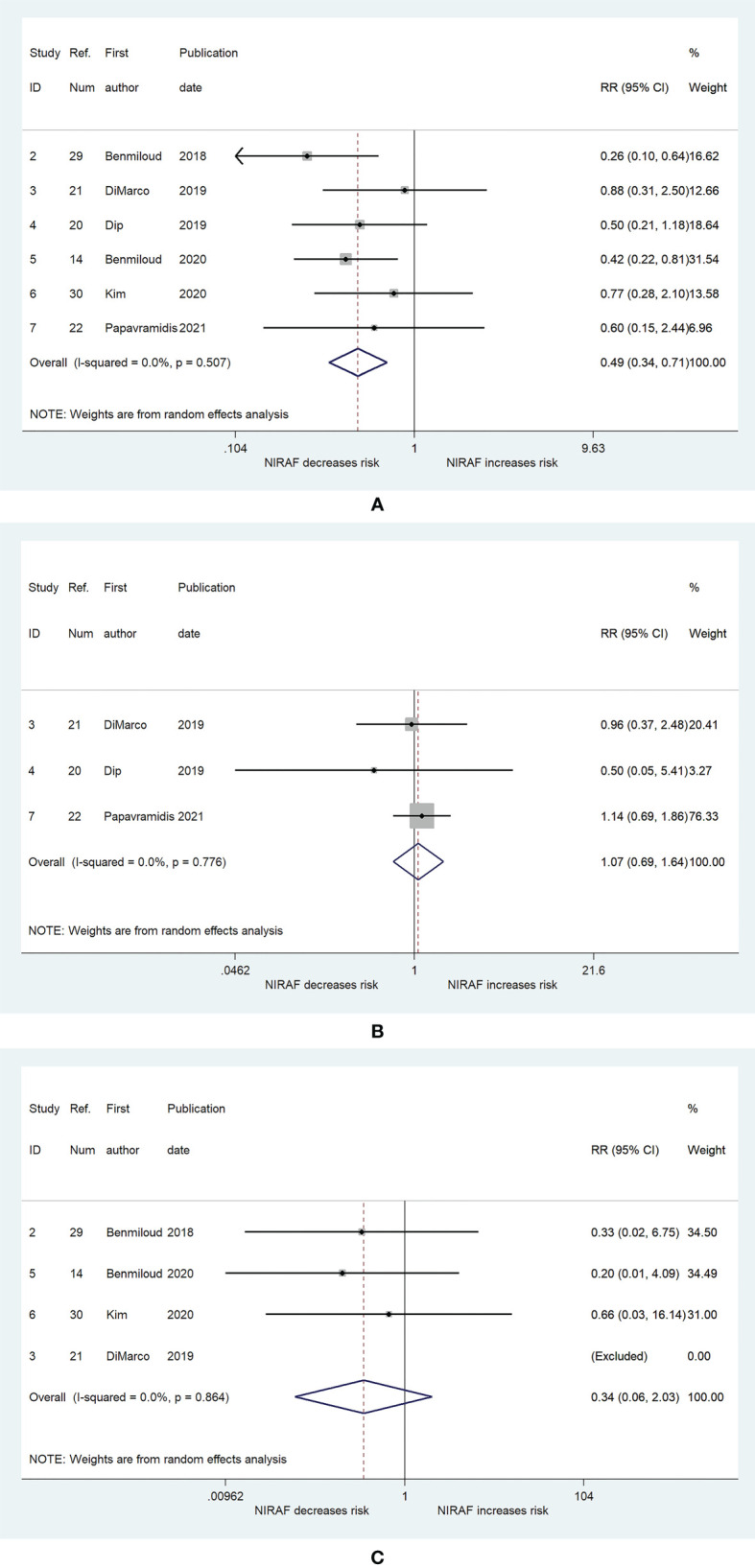
The relative risk for postoperative hypocalcemia. **(A)** Hypocalcemia at 1 day postoperatively. **(B)** Severe hypocalcemia at 1 day postoperatively. **(C)** hypocalcemia at 6 months postoperatively. The shaded areas represent the weight of the relevant study in pooling the results; the error bars represent the 95% confidence interval; the arrow indicate that the lower limits of the 95% confidence interval exceed the figure scale.

The results presented in **Figure 4** combined the RRs for the risk of hypocalcemia after total thyroidectomy. Six (14, 20–22, 29, 30) and four (14, 21, 29, 30) studies were used to generate the results of association between NIRAF and hypocalcemia at 1 day postoperatively and 6 months postoperatively, respectively. The incidence of hypocalcemia at 1 day postoperatively was lower in the NIRAF group than that in the naked eye group (RR = 0.49; 95% CI, 0.34–0.71, p < 0.001, **Figure 4A**), and no heterogeneity or publication bias was observed (I^2^ = 0%, p = 0.507, **Figure 4A**; Begg, p = 0.26; Egger, p = 0.375). However, compared with that in the naked eye group, the incidence of severe hypocalcemia, with which patients required calcium and vitamin D supplementation, at 1 day postoperatively, was not significantly different in the NIRAF group (RR = 1.07; 95% CI, 0.69–1.64, p = 0.765; I^2^ = 0%, p = 0.776, **Figure 4B**; Begg, p = 0.296; Egger, p = 0.299). There was also no significant difference in the incidence of hypocalcemia at 6 months postoperatively between the NIRAF group and the naked eye group (RR = 0.34; 95% CI, 0.06–2.03, p = 0.238; I^2^ = 0%, p = 0.864, **Figure 4C**; Begg, p > 0.99; Egger, p = 0.282).

## Discussion

The present meta-analysis demonstrated that NIRAF might help to identify PG and reduce PG autotransplantation and could assist in decreasing the incidence of inadvertently resected PG, thus lowering the incidence of hypocalcemia at 1 day postoperatively. However, NIRAF was not significantly associated with severe hypocalcemia at 1 day postoperatively and hypocalcemia at 6 months postoperatively.

PG identification is the first step in protecting the PG during thyroid surgery (31). Almost all of the present PG protection strategies are based on PG identification (11, 12, 15). Benmiloud et al. indicated that NIRAF helps to identify PGs (29). However, Dip et al. suggested that there was no significant difference in the number of identified PGs between the NIRAF group and the naked eye group (20). The reason for the different results might be due to the difference in the NIRAF use time. The former used NIRAF before dissection of the thyroid, while the latter used NIRAF after dissecting the thyroid. It is well known that thyroid dissection can reveal PG to the naked eye. This meta-analysis did not find that NIRAF was associated with an increased number of identified PGs, which might be attributed to the extensive experience of the chief surgeons who were all professional senior endocrine surgeons and identified almost all PGs. In other words, NIRAF was able to provide similar help in identifying PG as experienced endocrine surgeons, which would assist young surgeons in achieving thyroid surgery with a low incidence of hypoparathyroidism.

Inadvertently resected PG is a risk factor for postoperative transient and permanent hypoparathyroidism (32, 33). Therefore, it is important for PG function protection to identify them before resecting the specimens and find them out in the intraoperative specimens. According to this meta-analysis, NIRAF was associated with a lower incidence of inadvertently resected PG, which is consistent with the majority of studies (14, 22, 30). This phenomenon might be explained by the fact that NIRAF helped to identify more PGs, although the difference was not statistically significant.

PG autotransplantation is the exclusive method to protect the function of PGs, which cannot be preserved in site (11). Although the relationship between PG autotransplantation and permanent hypoparathyroidism is controversial, it is the consensus that PG autotransplantaton increases the incidence of postoperative transient hypoparathyroidism (7, 34, 35). This meta-analysis did not show a significant association between NIRAF and PG autotransplantation. Because the location and blood supply of PG, surgery method and surgical skill are also important influencing factors in preserving PG in site, besides PG identification. The small number of studies might be another reason for this result.

The incidence of postoperative hypocalcemia reflects the protective effect of the PG. Therefore, it is the primary evaluation index of protective ability. In the included studies, the incidence of hypocalcemia at 1 day postoperatively varied from 3.3% to 21.7%, which is consistent with previous reports (3–5). The meta-analysis demonstrated that NIRAF was associated with a decrease in the incidence of hypocalcemia at 1 day postoperatively. Less PGs were inadvertently resected in the NIRAF group, which might be responsible for this result. Another reason might be that NIRAF helped surgeons discover PGs earlier and thus added the probability of reducing PG damage. Several studies have confirmed that 36.9%–61.6% of PGs were identified by the NIRAF instrument before the surgeon saw them with the naked eye (14, 20, 30). However, the severe hypocalcemia at 1 day postoperatively was not associated with NIRAF according to the meta-analysis. Owing to the rich experience of the surgeons in these studies, the incidence of severe hypocalcemia at 1 day postoperatively might not be further decreased by using the NIRAF instrument.

In terms of hypocalcemia at 6 months postoperatively, the meta-analysis showed no effect of NIRAF. During the included studies, the maximal incidence of hypocalcemia at 6 months postoperatively and the largest sample size of the study was 1.69% and 300 patients, respectively. One study even showed that hypocalcemia did not occur at 6 months postoperatively in both the NIRAF and naked eye groups (21). The low incidence and small sample size of the study might not be sufficient to determine the difference brought by NIRAF. The experience and endeavor of the surgeons to prevent permanent hypocalcemia might also play a role in narrowing the difference. Su et al. suggested that the fourth PG did not significantly influence the overall PG function regardless of whether it was preserved in site, autotransplanted, or inadvertently resected, when three PGs were preserved in site (7).

Although there was no heterogeneity among the studies regarding the relationship between NIRAF and postoperative hypocalcemia, substantial heterogeneity was observed among the studies with respect to the relationship between NIRAF and PG identification, autotransplantation, and inadvertent resection. Heterogeneity was a major problem affecting the reliability of the pooled effect size in the meta-analysis. The following factors might have influenced the heterogeneity: (1) the reference standard consisted of experience and pathology in almost all the studies, which would be affected by subjectivity and thus could not be maintained completely consistent; (2) despite being professional thyroid surgeons, surgeons in different studies might still have differences in the ability to identify PG and the skills to preserve PG in site; (3) the surgical methods were not completely consistent; (4) measurement instruments and methods were not completely uniform; (5) the characteristics of the populations varied in different studies; (6) the confounding factors were different across these studies; and (7) the types of these studies were different, and thus, the quality of each study was not completely consistent.

This meta-analysis has several limitations. First, because the included studies consisted of a before–after study in the same patient, a historical controlled study, cohort studies, case–control studies, and randomized controlled trials, bias was inevitable. Second, the index related to serum parathyroid hormone, which is the direct indicator of the overall PG function, such as the incidence of hypoparathyroidism, the difference in serum parathyroid hormone levels before and after surgery, and the degree of decline in serum parathyroid hormone levels after surgery, was not collected and analyzed in these studies. Third, the heterogeneity was significant in the studies that explored the relationship between NIRAF and PG identification, autotransplantation, and inadvertent resection. Finally, although seven independent studies were included in this meta-analysis, the sample size was still limited, and the number of studies was smaller when a specific outcome analysis was performed.

## Conclusion

This meta-analysis demonstrates that NIRAF is significantly associated with a reduced risk of inadvertent parathyroid gland resection and hypocalcemia at 1 day postoperatively. The association between NIRAF and PG autotransplantation or hypocalcemia at 6 months postoperatively was not significant. Considering the limited number of studies, more studies are needed to explore the protective ability of NIRAF. Moreover, because serum parathyroid hormone is the direct indicator of PG function, future research should also report the effects on the indexes related to serum parathyroid hormone from NIRAF.

## Data Availability Statement

The original contributions presented in the study are included in the article/supplementary material. Further inquiries can be directed to the corresponding author.

## Author Contributions

Study conception and design: BW, C-RZ, HL, X-MY, and JW. Acquisition of data: BW and C-RZ. Analysis and interpretation of data: BW, C-RZ, and HL. Drafting of manuscript: BW and C-RZ. Critical revision: BW, C-RZ, HL, X-MY, and JW. Final approval of the version to be submitted: BW, C-RZ, HL, X-MY, and JW. All authors contributed to the article and approved the submitted version.

## Funding

BW was supported by a non-profit fund from China Health Promotion Foundation. JW was supported by a grant from Scientific Research Fund of the Department of Science and Technology of Chengdu City (2015-HM01-00376-SF) and Science and Technology Program of Science & Technology Department of Sichuan Province (2015JY0190). The funding bodies had no role in the conception of the study, in the collection, analysis, and interpretation of data, in writing the manuscript, and in the approval of the publication.

## Conflict of Interest

The authors declare that the research was conducted in the absence of any commercial or financial relationships that could be construed as a potential conflict of interest.

## Publisher’s Note

All claims expressed in this article are solely those of the authors and do not necessarily represent those of their affiliated organizations, or those of the publisher, the editors and the reviewers. Any product that may be evaluated in this article, or claim that may be made by its manufacturer, is not guaranteed or endorsed by the publisher.
